# CXCR4-Related Increase of Circulating Human Lymphoid Progenitors after Allogeneic Hematopoietic Stem Cell Transplantation

**DOI:** 10.1371/journal.pone.0091492

**Published:** 2014-03-12

**Authors:** Salomé Glauzy, Isabelle André-Schmutz, Jérôme Larghero, Sophie Ezine, Régis Peffault de Latour, Hélène Moins-Teisserenc, Sophie Servais, Marie Robin, Gérard Socié, Emmanuel Clave, Antoine Toubert

**Affiliations:** 1 Univ Paris Diderot, Sorbonne Paris Cité, Institut Universitaire d'Hématologie, Paris, France; 2 INSERM UMR1160, Paris, France; 3 Laboratoire d'Immunologie et d'Histocompatibilité, Hôpital Saint-Louis, AP-HP, Paris, France; 4 INSERM U768, Université Paris Descartes, Sorbonne Paris Cité, Institut Imagine, Paris, France; 5 Unité de Thérapie Cellulaire et CIC de Biothérapies, Hôpital Saint-Louis, AP-HP, Paris, France; 6 INSERM, Unité 1020, Paris, France; 7 Service d'Hématologie-Greffe de Moelle, Hôpital Saint-Louis, AP-HP, Paris, France; B.C. Cancer Agency, Canada

## Abstract

Immune recovery after profound lymphopenia is a major challenge in many clinical situations, such as allogeneic hematopoietic stem cell transplantation (allo-HSCT). Recovery depends, in a first step, on hematopoietic lymphoid progenitors production in the bone marrow (BM). In this study, we characterized CD34^+^Lin^−^CD10^+^ lymphoid progenitors in the peripheral blood of allo-HSCT patients. Our data demonstrate a strong recovery of this population 3 months after transplantation. This rebound was abolished in patients who developed acute graft-versus-host disease (aGVHD). A similar recovery profile was found for both CD24^+^ and CD24^−^ progenitor subpopulations. CD34^+^lin^−^CD10^+^CD24^−^ lymphoid progenitors sorted from allo-HSCT patients preserved their T cell potentiel according to *in vitro* T-cell differentiation assay and the expression profile of 22 genes involved in T-cell differentiation and homing. CD34^+^lin^−^CD10^+^CD24^−^ cells from patients without aGVHD had reduced CXCR4 gene expression, consistent with an enhanced egress from the BM. CCR7 gene expression was reduced in patients after allo-HSCT, as were its ligands CCL21 and CCL19. This reduction was particularly marked in patients with aGVHD, suggesting a possible impact on thymic homing. Thus, the data presented here identify this population as an important early step in T cell reconstitution in humans and so, an important target when seeking to enhance immune reconstitution.

## Introduction

In many clinical situations, such as intensive chemotherapy, acquired immunodeficiencies and allogeneic hematopoietic stem cell transplantation (allo-HSCT), immune recovery is a major challenge. Allo-HSCT is used to treat malignant and nonmalignant blood diseases, and remains the most widely used regenerative therapy. Accordingly, the profound and long-lasting immunodeficiency following this treatment leads to an increased risk of infection or relapse. Complete rescue of immune competence is closely linked to the recovery of de novo T-cell production in the thymus. This is a slow process depending on many factors, including patient age, disease status, source and composition of graft, type of conditioning, whether the donor is related or unrelated, human leukocyte antigen (HLA) mismatches, and the occurrence of graft-versus-host disease (GVHD) [1].

The thymus is the primary lymphoid organ responsible for T lymphocyte generation throughout life, its role is essential for post-lymphopenia reconstitution [2]–[4]. Two murine models have recently challenged the view that thymus function is exclusively dependent on the supply of bone marrow (BM)-derived progenitors [5], [6]. However, these studies involved animals with a defective functional progenitor supply. In human clinical situations, apart from with congenital T-cell immunodeficiencies, lymphoid progenitors are available. Thus, in most settings, T-cell immune reconstitution relies on the generation of lymphoid progenitors in the BM, their homing to the thymus for T-cell commitment and intrathymic differentiation. Despite increasing knowledge of the hierarchy of hematopoietic lineage determination in humans [7], no study has yet characterized lymphoid progenitors in the context of immune reconstitution in humans. Six et al. [8] characterized two progenitor populations: CD34^+^Lin^−^CD10^+^CD24^−^ cells, with a very low myeloid potential capable of giving rise to B, T and NK lymphocytes; and CD34^+^Lin^−^CD10^+^CD24^+^ cells, which are already committed to the B-cell lineage. The CD24^−^ population may represent thymus-seeding progenitors (TSP). It is found in cord blood (CB), BM, thymus and, importantly, it is detectable in peripheral blood throughout life. These cells express low levels of CD38 and may correspond to the multilymphoid progenitor population (MLP) described by Doulatov et al. [9]. MLPs are defined by their potential to give rise to B cells, T-cells and NK cells. In most cases, they also retain some potential towards the granulo-monocytic and dendritic cell lineages. More recently, data from gene expression profiles and functional analysis have shown that, in human BM, CD10^−^CD62L^hi^ progenitors are developmental intermediates between the multipotent CD34^+^Lin^−^CD38^−^ population and the lymphoid progenitor, or CD34^+^Lin^−^CD10^+^, population [10]. However, this study did not distinguish between CD34^+^Lin^−^CD10^+^CD24^−^ progenitors and the more prevalent B-cell-committed CD24^+^ population. The relationship between CD10^−^CD62L^hi^ and TSP remains to be elucidated.

In allo-HSCT, T-cell reconstitution in patients is impaired during acute GVHD (aGVHD). This disease could target the BM niche and hamper hematopoietic progenitor generation, TSP homing to the thymus, and even thymic function itself. In experimental murine models, aGVHD has been linked to both a reduction in thymic cellularity and destruction of the thymic architecture [11]–[13]. In the thymus, stromal and epithelial cells produce growth factors locally, as well as cytokines essential for thymocyte survival such as interleukin (IL)-7, stem cell factor (SCF), and Flt3L; or chemokines involved in T-cell precursor homing such as C-C chemokine ligand (CCL) 19, CCL21 and CCL25. In addition to thymic GVHD, murine models revealed the existence of an early BM GVHD, where the osteoblastic hematopoietic niche is targeted [14]. Therefore, aGVHD could reduce the number of multipotent precursor cells periodically released from the BM and migrating to the thymus. Indeed, Zlotoff et al. [15] showed that, in mice, a limited supply of hematopoietic progenitors to the thymus may impair T-cell recovery after allo-HSCT. In humans, we have shown that aGVHD transiently impairs thymic output in young patients after allo-HSCT [16]. aGVHD affected T-cell differentiation prior to TCR β-chain recombination, as shown by a decrease in T-cell receptor (TCR) β-chain rearrangement circles (TREC) in peripheral blood. T-cell progenitors have, as yet, to be characterized during T-cell recovery after allo-HSCT, and the consequences of aGVHD examined. This could help to better define which steps should be targeted to optimize T-cell immune reconstitution in immune compromised patients.

This article presents a snapshot analysis of cells between the time when they leave the BM and when the thymus is seeded. For the first time in humans and despite their very small number, we have characterized the recovery of circulating lymphoid progenitors, particularly the CD34^+^Lin^−^CD10^+^CD24^−^ population, in patients after allo-HSCT. We show that their quantitative recovery at 3 months post-transplant is affected by aGVHD and associated with downregulation of CXCR4 expression.

## Patients and Methods

### Patient characteristics

The study population consisted of 107 patients who received a non–T-cell–depleted allogeneic HSCT at the Bone Marrow Transplantation Unit, Hôpital Saint-Louis (Paris, France) between February 2002 and August 2012. Details on patients, donors and graft characteristics, as well as on transplantation outcome, are reported in Table 1. Acute GVHD was diagnosed and graded according to published criteria [17]. Nine of the 59 patients with grade 0-I aGVHD and 43 of the 48 patients with grade II–IV aGVHD were treated with corticosteroids. No significant difference was found between patient groups for sex ratio, GVH (0, I vs. II, III, IV), age, PB/BM, RIC (reduced intensity conditioning)/MAC (myeloablative conditioning) and diagnosis before allo-HSCT. Almost all patient were full donor chimera at month 3 as assesed by microsatellite analysis and only 1 patient had cells less than 92% of donor origin. Seventy-nine out of 107 patients were monitored for immune reconstitution by lymphocyte phenotyping and sjTREC content. Because of the small number of progenitor cells, different groups of patients were studied in the subsequent experiments: 39 for flow cytometry analysis, 22 for PCR analysis, 6 in OP9-ΔL1 co-culture and 40 to quantify plasma analytes. Peripheral blood samples (up to 25 mL) were collected at 3 months after transplant. Peripheral blood mononuclear cells (PBMCs) were separated on lymphocyte separation medium and either sorted immediately or frozen in Fetal Calf Serum (FCS), 10% DMSO for phenotypic analysis. As control, peripheral blood samples from healthy donors (HD) (n = 46) and cord blood (n = 8) were obtained from the Hôpital Saint-Louis Transfusion Center and Cord Blood Bank, respectively. These samples were treated according to the same protocols as patient samples. No significant difference was found between patients and HD in terms of age and sex ratio. The investigation was approved by the Committee on Medical Ethics of the Hôpital Saint-Louis, and written informed consent was obtained from all participants in accordance with the Declaration of Helsinki.

**Table 1:** BMT pairs, disease, and transplant characteristics.

	Acute GVHD	
CHARACTERISTICS	Gr 0-I	Gr II-IV
RECIPIENTS	n = 59	n = 48
Male/Female	40/19	27/21
Median age, y (range)	36 (6–66)	40 (7–66)
Positive CMV Serology	35 (59%)	27 (56%)
Underlying diagnosis		
- Chronic Leukemia	3 (5.1%)	6 (12.5%)
- Acute Leukemia	25 (42.4%)	25 (52.1%)
- Other malignant hemopathies	11 (18.6%)	6 (12.5%)
- Myelodysplasia	5 (8.5%)	7 (14.6%)
- Aplasia	15 (25.4%)	4 (8.3%)
**DONORS**		
Male/Female	30/29	25/23
Median age, y (range)	33 (5–65)	36 (21–66)
Female donor to male recipient	17 (29%)	13 (27%)
Positive CMV Serology	31 (53%)	30 (62.5%)
**TRANSPLANTATION**		
Donor		
- SIB	49 (83%)	37 (77%)
- MUD	10 (17%)	11 (23%)
Source of cells		
- PB	35 (59%)	27 (56%)
- BM	24 (41%)	21 (44%)
Conditioning		
- Nonmyeloablative	25 (42%)	16 (33%)
- TBI-based	17 (29%)	23 (48%)
- Busulfan-based	24 (41%)	19 (40%)
GVHD Prophylaxis		
- CSA	1 (2%)	0 (0%)
- CSA+MTX	29 (49%)	28 (58%)
- CSA+MMF	15 (25%)	14 (29%)
- CSA+Others	2 (3%)	1 (2%)
- Tacrolimus	1 (2%)	1 (2%)
Acute GVHD grades:		
- 0	46 (78%)	
- 1	13 (22%)	
- 2		35 (73%)
- 3		11 (23%)
- 4		2 (4%)

No significant difference was found between these two groups for the characteristics listed. BMT: bone marrow transplantation; SIB: HLA-matched sibling, MUD: HLA-matched unrelated donor; PB: peripheral blood; BM: bone marrow; TBI: Total body irradiation; CSA: cyclosporin A; MTX: Methotrexate; MMF: Mycophenolate mofetil; GVHD: Graft-versus-host disease.

### Flow cytometry analysis

All antibodies were from BD Biosciences, unless otherwise indicated. PBMC were thawed and at least 2×10^6^ cells were stained with monoclonal mouse anti-human CD34-allophycocyanin (APC; 8G12), monoclonal mouse anti-human CD24-fluorescein isothiocyanate (FITC; ML5) and monoclonal mouse anti-human CD10-phycoerythrin cyanine 7 (PE-Cy7; HI10a). The lineage (Lin) PE-conjugated antibody cocktail contained antibodies against CD2 (RPA-2.10), CD3 (UCHT1), CD4 (RPA-T4), CD8 (RPA-T8), CD13 (WM15), CD14 (M5E2), CD15 (HI98), CD16 (3G8), CD19 (HIB19), CD20 (2H7), CD33 (WM53), CD56 (B159), and CD235a (GA-R2). Cells were also stained with LIVE/DEAD Fixable Dead Cell Stain (aqua-fluorescent reactive dye, Invitrogen). Absolute numbers of total lymphocytes were determined using BD TruCOUNT Tubes. For immune reconstitution analysis, PBMC were thawed and stained with CD4-peridinin chlorophyll protein (PerCP), CD45RA-PE, CD19-PE, CD62L-FITC (Beckman Coulter) and CD27 (Pharmingen). Stained cells were analyzed on a FACS Canto II running with FACS DIVA software (BD Biosciences). Absolute counts of CD34^−^Lin^−^CD10^+^CD24^−^ and CD34^−^Lin^−^CD10^+^CD24^+^ cells were determined using the lymphocyte count. Data were analyzed for statistical significance by non-parametric Mann-Whitney test and Spearman correlation test.

### sjTREC quantification

PBMCs were separated on lymphocyte separation medium (Eurobio, Les Ulis, France), and 5 to 10×10^6^ cells were lysed and stored in TriReagent (Molecular Research Center, Cincinnati, OH). RNA and genomic DNA were then extracted from samples in line with manufacturer's instructions. sjTREC were quantified by real-time quantitative PCR (ABI PRISM7700; Applied Biosystems, Foster City, CA) as described [16]. Values were normalized for the genomic copy number, based on albumin gene quantification. Data were expressed per 150 000 PBMC, or the total number of TREC per cubic millimeter of blood was calculated using the absolute cell counts determined using the TruCOUNT system.

### Cell sorting

EDTA blood tubes were collected, and PBMCs were separated on lymphocyte separation medium. CD34^+^ cells were enriched using CD34 MicroBead Kit (MACS, Miltenyi Biotec) before staining for flow cytometry analysis as described above. The CD34^−^ fraction was counted and frozen in FCS, 10% DMSO or lysed in TriReagent. The stained CD34^+^ fraction was sorted on a FACS ARIA (BD Biosciences) to isolate CD34^+^lin^−^CD10^+^CD24^−^ cells. Up to 400 CD34^+^lin^−^CD10^+^CD24^−^ cells and CD34^+^Lin^−^CD10^−^ were collected in 1.5 ml tubes containing 60 μL of RLT Buffer for multiplex PCR analysis or CD34^+^lin^−^CD10^+^CD24^−^ cells were sorted and seeded at 1, 5, 10, 20, 40 and 100 cells on OP9-hDelta1-coated 96-well plates for limiting dilution assay.

### *In vitro* T-cell differentiation limiting dilution assay

T lymphoid potential was tested by plating candidate progenitors on OP9 stromal cells. The stromal cells were transduced with a replication-defective retrovirus containing a double cassette expressing the human Notch ligand Delta1 (hDelta1) and GFP [8]. All cocultures were performed in complete medium (αMEM (Gibco, Invitrogen) supplemented with 20% FCS HyClone (ThermoSCIENTIFIC), 1% penicillin, 1% streptomycin, 5 ng/ml Flt3L, 10 ng/ml SCF and 2 ng/ml IL-7 (all from R&D Systems)). After 3 weeks, wells containing CD5^+^CD7^+^ cells were identified by flow cytometry. Data are presented as the percentage of negative wells against the Log_10_ of the initial number of cells plated per well. The proportion of lymphoid precursors was calculated by linear regression based on a Poisson distribution. Data were analyzed for statistical significance using a non-parametric Mann-Whitney test. For ethical and methodological reasons, patients with severe aGVHD could not be tested. Patient blood samples measured 30–35 mL, whereas HD provided 100 mL. The high volume of blood from healthy donors compensated the low frequency of CD34^+^lin^−^CD10^+^CD24^−^ cells. In patients without aGVHD, the higher frequency of these cells permitted us to sort them despite the small amount of blood. In patient with severe aGVHD their lower frequency associated with the small amount of blood could not allow us to sort enough CD34^+^lin^−^CD10^+^CD24^−^ cells for the assay.

### Quantitative real-time PCR

After cell sorting, up to 400 CD34^+^lin^−^CD10^+^CD24^−^ and CD34^+^Lin^−^CD10^−^ were lysed in TriReagent. Samples were used to extract RNA according to manufacturer's instructions. RNA was reverse transcribed (with random hexamers) and amplified (with specific primer, Table S1) using a GeneAmp RNA PCR Kit (Applied Biosystems). For this amplification, the genes studied were split into two groups: CCR7, CCR9, CXCR4, CD44, ITGα4, PSGL1 and RPL27 in the first one; and Notch1, HES1, cKIT, IL7Rα, CD3ε, CD4, CD8b, CD38, GATA3, Ikaros, RAG1, RORC, cMYB, EBF1, GATA1, LMO2 and RPL27 in the other. Quantitative amplification was performed for each gene in SYBR Green on a 7500 Fast Real-Time PCR System (Applied Biosystems). The same primer couple was used for multiplex amplification and simplex real-time PCR. Data were expressed as expression relative to the RPL27 housekeeping gene, and analyzed for statistical significance by a non-parametric Mann-Whitney test and using Principal Component Analysis (PCA) on SPSS. PCA is a mathematical procedure that attempts to explain as much of the total variation in the data set using as few factors (principle components) as possible.

### Quantification of cytokine and chemokine concentration

Plasma was collected from EDTA blood samples. CCL25 concentration was determined with a human CCL25/TECK DuoSet ELISA (R&D System). IL-7 concentration was determined using Quantikine HS ELISA human IL-7 (R&D System). Plates were read on a Biochrom Anthos Zenyth 340 s. SDF-1α+β, 6Ckine (CCL21) and CCL19 concentrations were determined with MILLIPLEX MAP KIT Human Cytokine/Chemokine Panel II and III (MILLIPORE). Plates were read on a LABScan 100 (Luminex). All analytes were measured in plasma from 20 HD and 40 patients 3 months after HSCT (including 20 with grade II to IV aGVHD). Data were analyzed by non-parametric Mann-Whitney test.

## Results

### Circulating lymphoid progenitor rebound after allo-HSCT in humans is decreased by aGVHD

**Figure 1:**
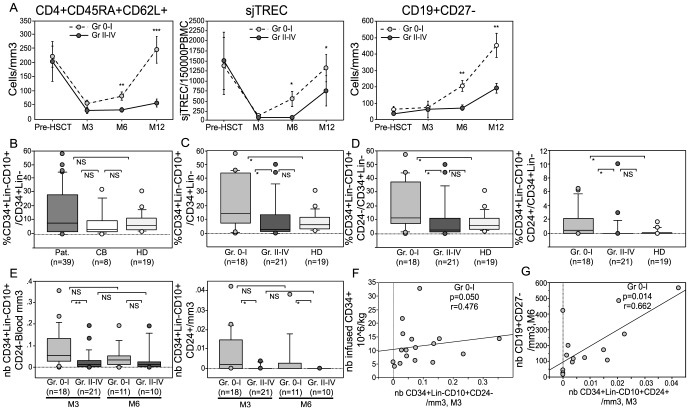
Lymphoid progenitors are detected in peripheral blood after allo-HSCT and are increased in the absence of aGVHD. (A) Naïve T-cell and B-cell immune recovery was measured by CD4^+^CD45RA^+^CD62L^+^ and CD19^+^CD27^−^ staining and flow cytometry analysis and thymic output was determined by quantitative PCR for sjTREC in 79 patients with (closed circles) and without (open circles) aGVHD from time pre-HSCT to M12 (12 Months post-HSCT). (B) Mononuclear cells from cord blood (CB) and peripheral blood were stained with a combination of lineage (Lin) markers and anti-CD34, anti-CD10, and anti-CD24 antibodies. Percentage of CD34^+^lin^−^CD10^+^ progenitors/CD34^+^Lin^−^ cells in CB, peripheral blood from HD and HSCT patients 3 months post-graft. (C) Percentage of CD34^+^lin^−^CD10^+^ progenitors/CD34^+^Lin^−^ cells for HSCT patients with grade 0-I (Gr 0-I) or II-IV (Gr II-IV) aGVHD. (D) Percentage of CD34^+^Lin^−^CD10^+^CD24^−^/CD34^+^Lin^−^ and of CD34^+^Lin^−^CD10^+^CD24^+^/CD34^+^Lin^−^ in Gr 0-I *vs*. Gr II-IV patients and *vs.* HD. (E) Absolute numbers of CD34^+^Lin^−^CD10^+^CD24^−^ and CD34^+^lin^−^CD10^+^CD24^+^ in HSCT patients at months 3 (M3) and 6 (M6). (F) Correlation between absolute numbers of CD34^+^Lin^−^CD10^+^CD24^−^ and the number of CD34^+^ cells in the graft, normalized by recipient weight. Correlation is positive in the absence of aGVHD, but not with severe aGVHD (*Spearman test*). (G) Correlation between absolute numbers of CD34^+^Lin^−^CD10^+^CD24^+^ B progenitors at month 3 and the number of naïve B cells at month 6. (Spearman test).**p<0.01, *p<0.05, NS: Not Significant (Mann-Whitney).

Naïve T-cell reconstitution was analyzed in a cohort of 79 patients (see Patients and Methods and Table 1) using flow cytometry to track CD4^+^CD45RA^+^CD62L^+^ cells; thymic output was measured by quantitative PCR for sjTREC. As we reported previously [16], patients with grade II to IV aGVHD showed a decreased reconstitution at 3 and 6 months after allo-HSCT. We also observed a delay in naïve CD19^+^CD27^−^ B-cell reconstitution with aGVHD (Fig. 1A). Based on these observations, month 3 was chosen as the best time point at which to assess the CD34^+^lin^−^CD10^+^CD24^−^ population after allo-HSCT.

Circulating progenitors were quantified by flow cytometry in a subgroup of 39 allo-HSCT patients, in 19 age-matched healthy donors (HD), and in 8 cord blood (CB) samples (Fig. S1). Median percentage and absolute number of CD34+ were 0.131%CD34^+^/Living cells, Range [0.007-0.909] and 1.4 CD34^+^/blood mm^3^, Range [0.1 à 16], respectively. No differences were found between patients and HD in terms of frequency for either CD34^+^ or CD34^+^Lin^−^ cells (Data not shown). Lymphoid progenitors characterized as CD34^+^Lin^−^CD10^+^ cells could be detected throughout the postnatal period [8]. They have a very limited myeloid potential when cultured on MS5 stromal cells or in methylcellulose colony formation assay (data not shown). The percentage of CD34^+^Lin^−^CD10^+^ cells within the CD34^+^Lin^−^ population was not significantly different between CB, HD or allo-HSCT patients (%CD34^+^Lin^−^CD10^+^/CD34^+^Lin^−^ = 7 [0-58] and CD34^+^Lin^−^CD10^+^/blood mm3 = 0.03 [0.00 – 0.36]). However, the percentage of these cells was very variable among patients (Fig. 1B). Indeed, patients without aGVHD or with grade I aGVHD had a significantly higher proportion of CD34^+^Lin^−^CD10^+^/CD34^+^Lin^−^ cells than patients with aGVHD grade II, III or IV (p = 0.024) or HD (p = 0.033) (Fig. 1C).

A CD24^+^ B-cell-restricted subpopulation and a CD34^+^Lin^−^CD10^+^CD24^−^ subset can be distinguished within the postnatal CD34^+^Lin^−^CD10^+^ progenitor population based on CD24 expression [8]. In our samples, the CD34^+^Lin^−^CD10^+^CD24^+^ subpopulation was found in CB, but was extremely rare in peripheral blood, detectable in only 5 out of the 19 HD samples. In patients at 3 months after HSCT without aGVHD, we observed a rebound of both CD24^−^ and CD24^+^ progenitor subpopulations with a significant increase in their percentages reaching values above HD (Fig. 1D). Absolute cell counts showed that the number of T/B/NK (CD34^+^Lin^−^CD10^+^CD24^−^) and B-cell committed (CD34^+^Lin^−^CD10^+^CD24^+^) progenitors decreased with increasing aGVHD severity (Fig. S2). Six months after transplantation, the median number of both CD24^+^ and CD24^−^ subsets had decreased (Fig. 1E).

Importantly, examining other pre-transplant parameters usually associated with aGVHD, no effect of recipients' age or conditioning regimen on CD34^+^Lin^−^CD10^+^, CD34^+^Lin^−^CD10^+^CD24^−^ and CD34^+^Lin^−^CD10^+^CD24^+^ counts was observed. The source of grafted cells (BM or mobilized peripheral blood stem cells) had a mild impact on CD34^+^Lin^−^CD10^+^ and CD34^+^Lin^−^CD10^+^CD24^−^ cell numbers, but in multivariate analysis including age, source of cells, and conditioning regimen, aGVHD was the only significant factor identified (p = 0.022 and p = 0.034, Table 2). Also, the grafted CD34^+^ cells dose is predictive of survival, posttransplant morbidity, and rate of hematologic recovery after allo-HSCT [18], [19]. Accordingly, in our study, there was a significant correlation between the number of CD34^+^ cells in the graft and the number of CD34^+^lin^−^CD10^+^CD24^−^ cells in peripheral blood at 3 months. This correlation was not seen in presence of severe aGVHD (Fig. 1F), suggesting that severe allogeneic BM reactions affect the supply of lymphoid progenitors to the periphery. In addition, another correlation was found between absolute numbers of B-cell-committed CD34^+^Lin^−^CD10^+^CD24^+^ progenitors at 3 months and total CD19^+^ cells, as well as naïve CD19^+^CD27^−^ B cells at 6 months after allo-HSCT (p = 0.014 and p = 0.014 respectively, Fig. 1G). This correlation was also only significant in the absence of aGVHD. Overall, at 3 months after allo-HSCT, we observed that the rebound of circulating lymphoid progenitors was abrogated in patients presenting severe aGVHD.

**Table 2:** Univariate and multivariate analysis of factors influencing CD34^+^Lin^−^CD10^+^ and TSP recovery after HSCT.

			CD34^+^Lin^−^CD10^+^			CD34^+^Lin^−^CD10^+^CD24^−^		
Factors		N	Median Nb/blood mm3 (Range)	Univariate analysis p	Multivariate analysis p	Median (Range)	Univariate analysis p	Multivariate analysis p
aGVHD	0-I	18	0.056 (0–0.362)	**0.003**	**0.022**	0.054 (0–0.354)	**0.005**	**0.034**
	II–IV	21	0.014 (0–0.194)			0.014 (0–0.194)		
Age	<25 y	10	0.039 (0–0.197)	0.334	0.846	0.026 (0–0.154)	0.421	0.763
	>25 y	29	0.027 (0–0.362)			0.014 (0–0.354)		
Stem cell source	BM	20	0.039 (0–0.236)	**0.025**	0.380	0.034 (0–0.235)	**0.028**	0.390
	PB	19	0.014 (0–0.362)			0.014 (0–0.354)		
Myeloablative conditioning	Yes	28	0.030 (0–0.236)	0.595	0.392	0.029 (0–0.235)	0.595	0.419
	No	11	0.027 (0–0.362)			0.027 (0–0.354)		

Statistical significance was tested using a non-parametric Mann-Whitney test for univariate data and using ANOVA for multivariate analysis.

### CD34^+^Lin^−^CD10^+^CD24^−^ progenitors display functional features of T-cell progenitors after allo-HSCT

**Figure 2:**
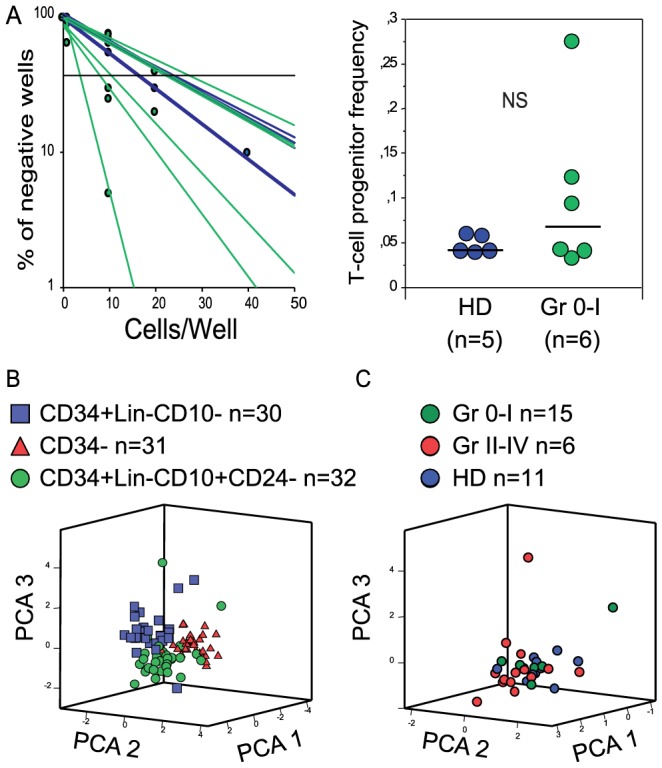
CD34^+^lin^−^CD10^+^CD24^−^ progenitors display functional features of T-cell progenitors after allo-HSCT. (A) Limiting dilution assay analysis on OP9-DL1 with CD34^+^Lin^−^CD10^+^CD24^−^ cells sorted from peripheral blood of healthy donors (HD) and at day 100 post allo-HSCT in patients without aGVHD, or with grade I aGVHD (Gr 0-1). *NS: Not Significant (Mann-Whitney)*. (B) Principal Component Analysis (PCA) of CD34^+^Lin^−^CD10^+^CD24^−^ (circles), CD34^+^Lin^−^CD10^−^ cells (squares) and CD34^−^ cells (triangles) from 11 HD and 21 allo-HSCT patients using a panel of 22 genes (see Materiel & Methods). (C) PCA of CD34^+^Lin^−^CD10^+^CD24^−^ from healthy donors (HD, open circles) or patients with (green filled circles) or without severe aGVHD (red filled circles).

The next step was to ensure that this increased circulating progenitor population detected after transplantation was truly T cell committed. To this aim, CD34^+^Lin^−^CD10^+^CD24^−^ from HD and patients without aGVHD were sorted and cultivated on the OP9-DL1 stromal cell line in the presence of Flt3L, IL-7 and SCF to check their T-cell differentiation potential [20]. CD34^+^lin^−^CD10^+^CD24^−^ cells from patients with severe aGVHD could not be assessed in this assay due to the small volume of blood (<25 ml) available for ethical reasons. In both HD and patients, FACS sorted cells effectively gave rise to T-cells in this system. Using limiting dilution, we found a similar frequency of T-cell progenitor between HD (mean = 1/19.3; n = 5) and patients without aGVHD (mean = 1/12.4; n = 6) (Fig. 2A). In conclusion, the rebound of circulating CD34^+^lin^−^CD10^+^CD24^−^ cells at 3 months after HSCT was accompanied by a sustained T-cell potential.

The gene expression profile was then assessed in two multiplex assays. Genes implicated in progenitor T cell differentiation, 22 genes in total, were screened using a quantitative gene expression method on very limited cell numbers [21]. The 22 genes analyzed were: the chemokine receptors *CCR7* and *CCR9; CXCR4*, a chemokine receptor which plays a role in progenitor egress from the BM; *CD44*, *ITGα4* and *PSGL1*, adhesion molecules particularly involved in adhesion between progenitor and thymic cells [22]; the cytokine receptors *IL7Rα* and *cKIT*; actors of the Notch pathway, *Notch1* and *HES1*; several transcription factors preferentially found in stem cells (*LMO2, cmyb*), progenitors of B cells (*EBF1*), T-cells (*GATA3, RORC, IKAROS*) or erythroid cells (*GATA1*) and finally some markers of the T lineage such as *CD3ε*, *CD4*, *CD8*, *CD38* and *RAG1*. Cells from 22 patients and 11 HD were isolated by FACS, then the real-time PCR gene expression median value for CD34^+^Lin^−^CD10^−^ cells was compared to that for CD34^+^Lin^−^CD10^−^ cells as a control of non-lymphoid primed progenitors, and to that for CD34^−^ cells (Table S2). Global gene expression was displayed using Principal Component Analysis (PCA) which factorizes the total variability in gene expression. This analysis efficiently discriminated between CD34^+^lin^−^CD10^+^CD24^−^ and the two other cell subpopulations (Fig. 2B). According to PCA, CD34^+^lin^−^CD10^+^CD24^−^ cells from HD and patients with or without aGVHD clustered together (Fig. 2C).

Thus, both *in vitro* differentiation on OP9-DL1 stromal cells and gene expression profiling showed the CD34^+^Lin^−^CD10^+^CD24^−^ population have an effective T-cell potential in the context of allo-HSCT.

### CXCR4 and CXCL12 expression profiles are consistent with progenitor egress from the bone marrow and are altered in aGVHD

**Figure 3:**
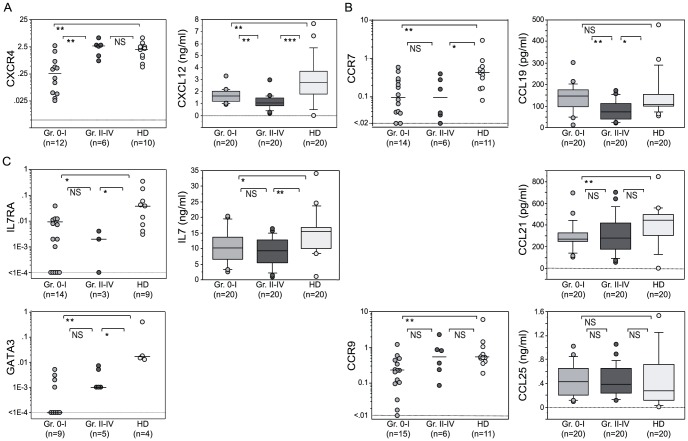
Allo-HSCT and aGVHD effects on CD34^+^Lin^−^CD10^+^CD24^−^ cells gene expression and plasma concentrations of chemokines and cytokines are associated with alterations to BM egress, thymic homing and T-cell commitment. (A) Relative CD34^+^Lin^−^CD10^+^CD24^−^ mRNA expression by quantitative RT-PCR of CXCR4 and plasma concentration of CXCL12. (B) RT-PCR quantification of CCR7, CCR9 and plasmatic dosage of their respective ligands CCL19, CCL21 and CCL25. (C) RT-PCR quantification of IL7RA and GATA3 and plasmatic dosage of IL-7 in healthy donors (HD, white boxes/circles), patients with grade II-IV aGVHD (Gr II-IV, black boxes/circles), patients with grade 0-I aGVHD (Gr 0-1, grey boxes/circles). *p<0.05, **p<0.01, ***p<0.001, NS: Not Significant (Mann-Whitney).

To get a further mechanistic insight into the CD34^+^lin^−^CD10^+^CD24^−^ increase, we examined the expression levels of genes involved in progenitor mobilization and homing, and the levels of chemokines in the plasma. We first studied the CXCR4/CXCL12 axis which plays an important role in retaining HSC and progenitors in the BM [23], [24]. Our results for patients without aGVHD showed a significant reduction in CXCR4 mRNA expression levels in CD34^+^lin^−^CD10^+^CD24^−^ cells. Plasma CXCL12 concentration was also reduced in all patients. This is consistent with disruption of CXCL12/CXCR4 signaling and release of lymphoid progenitors into the periphery (Fig. 3A). Patients with severe aGVHD have significantly lower plasma CXCL12 levels than patients without aGVHD (Fig. 3A) due to a reduced production or a higher destruction rate of the chemokine.

### Thymic homing and T-cell commitment could be altered after allo-HSCT, especially with severe aGVHD

Several molecules have been shown to play a role in thymus colonization in mice. These include CCR7 and its ligand (CCL19/21), CCR9 and its ligand (CCL25) [25], [26], and PSGL1 [27]. In humans, the mechanisms of thymus colonization have been less studied, but similar chemokines may be involved, at least during the fetal and perinatal periods [8], [10], [28]. In our study, whereas PSGL1 expression was not modified (Data not shown), CCR7 and CCR9 mRNA expression was reduced after allo-HSCT, even in the absence of aGVHD. The plasma concentration of the CCR9 ligand, CCL25, was not modified. Conversely, CCL19 and CCL21 concentrations were significantly reduced in patients after allo-HSCT. CCL19 was further reduced in patients presenting severe aGVHD (Fig. 3B).

The majority of T-cell commitment markers (CD3ε, IKAROS, RAG1) were not modified by allo-HSCT or GVHD. IL7 serum concentration is reported as maximum right after the transplant conditioning regimen and IL7 level at days +7, +14 [29] or +30 [30] has been associated with aGVHD incidence. However, in this study at day +90, GATA-3 and IL-7Rα mRNA expression, together with IL7 plasma concentrations, were significantly reduced in all patients compared to HD. These changes were independent of aGVHD status (Fig 3C).

## Discussion

The initial report of a rebound in thymic activity after intensive chemotherapy in children [31] has been extended to other settings in adults including autologous [32] and allogeneic [3] HSCT and appears to be crucial in the regeneration of peripheral T-cell populations. Thymic-dependent T-cell reconstitution is a slow process after allo-HSCT in humans which can be evaluated phenotypically, by TREC quantification and TCR diversity analysis [4]. Immune recovery is further impeded in humans during the aGVHD immune reaction [1]. Whereas the impact of aGVHD on thymic function has been documented [16], there is no data yet on the characteristics of lymphoid progenitors in the condition of human allo-HSCT and aGVHD.

Our findings show in humans that immune reconstitution needs to be considered in a global perspective integrating progenitor supply by the bone marrow and, for T cells, their competence in thymic homing and T-cell differentiation. It seems striking to us that the circulating progenitors rebound we detected 3 months after transplantation was concomitant to the reduced CXCR4 expression in these cells and to a decreased CXCL12 plasmatic concentration. CXCL12, or stromal cell-derived factor 1a (SDF-1a), is constitutively produced at high levels in the BM by stromal cells where it maintains HSC in a quiescent state. It is also required for their retention in the BM [23], [24]. Disruption of the interaction between CXCL12 and its receptor, CXCR4, results in the mobilization of cells from the BM and their release into the blood. CXCR4 antagonists, in particular AMD3100, have been used to mobilize HSCs for HSCT in humans [33]. Disruption of CXCL12/CXCR4 signaling is also a key step in HSC mobilization by G-CSF [34], [35].This lead us to suspect a causative role of CXCR4/CXCL12 in the lymphoid progenitors rebound during the profound lymphopenia observed after allo-HSCT. Whether this could apply to other situations of lymphopenia and myelosuppression, during intensive chemotherapy for instance, will deserve further studies. In the BM, CXCL12 is produced by osteoblast precursors and CXCL12-abundant reticular cells (CAR), a stromal perivascular population involved in HSC and B lymphoid progenitor maintenance [23]. Some recent evidence in mice indicates that early lymphoid progenitors occupy a distinct endosteal niche not shared by HSC [36], [37]. Acute GVHD may alter this microenvironment and the capacity to produce CXCL12. In line with this, in a murine model, osteoblasts were found to be the main target of aGVHD in BM [14]. CXCL12 degradation could also explain the reduced levels of this chemokine in the periphery, especially with severe aGVHD, as CXCL12 is extremely sensitive to proteolysis [35]. We believe that, in the absence of aGVHD, low CXCL12 levels favor proliferation and egress of committed progenitors from the BM as part of a regenerative mechanism to correct for lymphopenia. This hypothesis is supported by murine models of myelosuppression [24]. In patients presenting aGVHD, due to stromal injury and possibly also to therapies such as corticosteroids, a further decrease of CXCL12, together with disruption of the BM niche, could alter this process [38]. However, patient with grade I aGVHD that don't receive corticosteroid, had already less progenitor cells than patients without aGVHD (Fig. S2), arguing for a direct effect of the disease.

A second finding was the potential limitations in thymus seeding properties of CD34^+^lin^−^CD10^+^CD24^−^ progenitors in allo-HSCT and especially aGVHD. Together with the pronounced decrease in CCR7 expression, reduced plasma levels of ligands, especially CCL19, could further impede progenitor homing to the thymus. In the thymus, CCL19 and CCL21 have been detected in the medulla, the corticomedullary junction and in several cell types scattered throughout the cortex. CCL19 expression is widely associated with blood vessels [39] and with the vascular endothelium, lymphocyte migration to peripheral lymphoid organs being dependant on this molecule. The reduced CCL19 expression observed with GVHD could indicate alterations of the thymic epithelium and/or the vascular endothelium [40]. We also showed defects in T-cell commitment potential after allo-HSCT. GATA-3 is required for early T lineage progenitor development and probably controls the differentiation of TSP into Early Thymic Progenitors [41]. IL-7 plays critical and non-redundant roles in both T-cell lymphopoiesis and in maintaining and restoring peripheral T-cell homeostasis and early T-cell development [42]. These data together suggest that the homing properties of TSP to the thymus and their T-cell differentiation potential may be limited after allo-HSCT in adults, and that this limitation may be further enhanced in aGVHD.

In total, we show in humans a physiological rebound of circulating lymphoid progenitors after allo-HSCT. This rebound is impaired by aGVHD supporting BM stromal injury as the initial mechanism impacting immune recovery. In terms of therapeutic consequences, attempts should be made to protect the BM stromal microenvironment, and especially the endosteal niches where early lymphoid progenitors are found [36], [37]. Radiation-resistant myeloid cells, by limiting ROS production, may play a protective role in this zone under stress condition [43]. Finally, Cellular Therapy approaches have been developed to bypass the delivery of lymphoid progenitors from the BM by using adoptive transfer of *in vitro*-generated T-cell progenitors [44]. However, these approaches may also be hampered by an altered thymic environment when used in clinical settings.

## Supporting Information

Figure S1 Representative staining of lymphoid progenitors.
(PDF)

Figure S2 Absolute numbers of lymphoid progenitors correlate with aGVHD severity.
(PDF)

Table S1 Specific primer used for multiplex amplification and simplex real time PCR.
(PDF)

Table S2 Median or relative quantity to RPL27 for each gene.
(PDF)
