# Supplementary material for: CXCR4-Related Increase of Circulating Human Lymphoid Progenitors after Allogeneic Hematopoietic Stem Cell Transplantation
Source: PLoS One. 2014 Mar 12;9(3):e91492. doi: 10.1371/journal.pone.0091492 (PMC3951398; doi:10.1371/journal.pone.0091492)
Supplement: Figure S1 — Representative staining of lymphoid progenitors. (PDF) [file pone.0091492.s001.pdf]

Figure S1: Representative staining of lymphoid progenitors

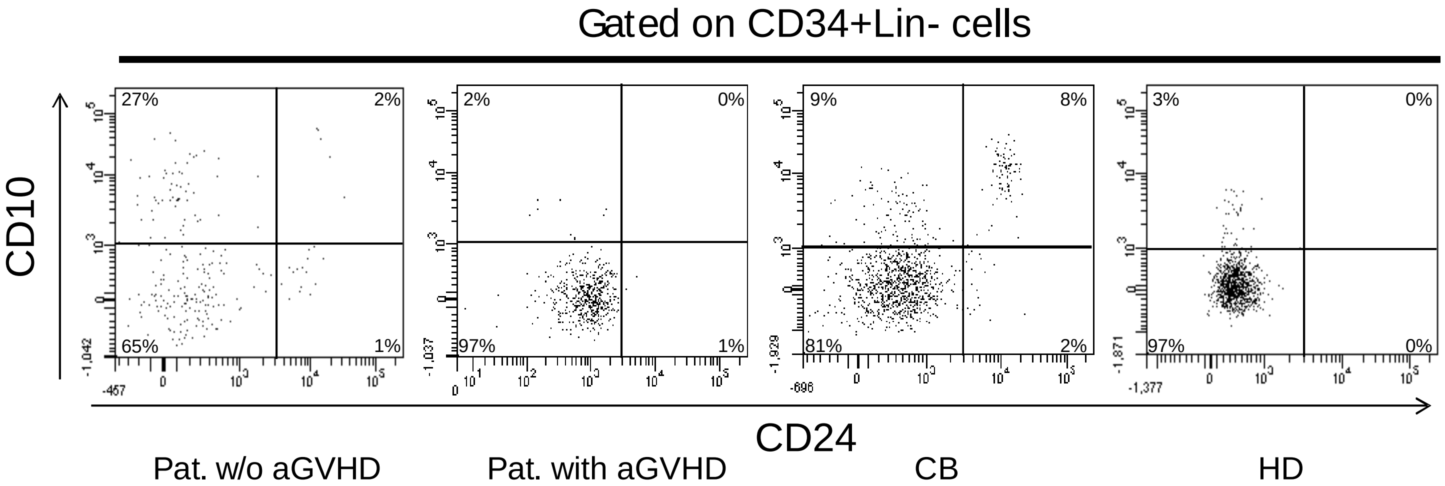

Mononuclear cells from cord blood (CB) and peripheral blood from healthy donors (HD) or patients, 3 months after allo-HSCT, with (Pat. with aGVHD) or without (Pat. w/o aGVHD) aGVHD were stained with a combination of lineage (Lin) markers and anti-CD34, anti-CD10 and anti-CD24 antibodies
