# Supplementary material for: CXCR4-Related Increase of Circulating Human Lymphoid Progenitors after Allogeneic Hematopoietic Stem Cell Transplantation
Source: PLoS One. 2014 Mar 12;9(3):e91492. doi: 10.1371/journal.pone.0091492 (PMC3951398; doi:10.1371/journal.pone.0091492)
Supplement: Table S1 — Specific primer used for multiplex amplification and simplex real time PCR. (PDF) [file pone.0091492.s003.pdf]

Table SI: Specific primer used for multiplex amplification and simplex real time PCR.

GENE	Forward primer	Reverse primer
CCR7	CAGCCTTCCTGTGTGGTTTT	TCCGTGACCTCATCTTGACA
CCR9	CACCCACAGACTTCACAAGC	CTCGCAAACTGCCTGACAT
CD3ε	GGCCTCTGCCTCTTATCAGTT	CCAGGATACTGAGGGCATGT
CD4	GCCTCCTGCTTTTCATTGG	CTGAAACCGGTGAGGACACT
CD8B	AAAGGGACTATCCACGGTGA	CCGACGATCATGCAGAAGTA
CD38	ACCTCACATGGTGTGGTGAA	ACCACATCACAGGCAGCTT
CD44	CGGACACCATGGACAAGTTT	CACGTGGAATACACCTGCAA
CKIT	CATTCCCCAAACCTGAACAC	CCTCCTTCGGTGCCTTTTA
CMYB	GACTATGATGGGCTGCTTCC	GCACTGCACATCTGTTCGAT
CXCR4	CAGCAGGTAGCAAAGTGACG	ATAGTCCCCTGAGCCCATTT
EBF1	GAAGCCAACAGCGAAAAGAC	ATGAGGCGCACGTAGAAATC
GATA1	AGAAGCGCCTGATTGTCAGT	CGGTTCACCTGGTGTAGCTT
GATA3	GTCCTGTGCGAACTGTCAGA	CTGGATGCCTTCCTTCTTCA
HES1	CCAAAGACAGCATCTGAGCA	CCGCGAGCTATCTTTCTTCA
IKAROS	CCAATGTGCTCATGGTTCAC	GGAATGCAGCTTGATGTGC
IL7Rα	TATCGTATGGCCCAGTCTCC	GAATCTGGCAGTCCAGGAAA
ITGα4	AACACGCTGTTCGGCTACTC	CTTTCCGATCCTGCATCTGT
LMO2	CGGCGCCTCTACTACAAACT	GTCTTTCACCCGCATTGTC
NOTCH1	CAACGCCTACCTCTGCTTCT	ACTCGTAGCCATCGATCTTGTC
PSGL1	GGCTGGGACCTTGTCACTAA	CCAGTAGGATCAGCAACAGGA
RAG1	CAGTCCATGGTCCTGTGGAT	CGAGTCAACATCTGCCTTCA
RORC	CAGCGCTCCAACATCTTCT	CGAACTCCACCACGTACTGA
RPL27	CCCTTGGACAAAACTGTCGT	ACCGCAGTTTCTGGAAGAAC
