# Supplementary material for: CXCR4-Related Increase of Circulating Human Lymphoid Progenitors after Allogeneic Hematopoietic Stem Cell Transplantation
Source: PLoS One. 2014 Mar 12;9(3):e91492. doi: 10.1371/journal.pone.0091492 (PMC3951398; doi:10.1371/journal.pone.0091492)
Supplement: Table S2 — Median or relative quantity to RPL27 for each gene. (PDF) [file pone.0091492.s004.pdf]

## Table SII: Median of relative quantity to RPL27 for each gene.

| MEDIANE OF RELATIVE QUANTITY TO RPL27 | CD34$^+$lin$^-$CD10$^+$CD24$^-$ | | | CD34$^+$lin$^-$CD10$^-$ | | | CD34$^-$ | | |
|---|---|---|---|---|---|---|---|---|---|
| | Gr 0-I | Gr II-IV | HD | Gr 0-I | Gr II-IV | HD | Gr 0-I | Gr II-IV | HD |
| CCR7 | 0,097 (0,000-0,581) | 0,096 (0,000-0,397) | 0,428 (0,080-2,981) | 0,009 (0,003-0,083) | 0,009 (0,000-0,314) | 0,004 (0,000-10,635) | 0,029 (0,000-0,547) | 0,046 (0,000-0,218) | 0,106 (0,020-4,689) |
| CCR9 | 0,226 (0,000-1,199) | 0,581 (0,088-2,306) | 0,521 (0,194-6,071) | 0,002 (0,000-0,179) | 0,007 (0,000-0,176) | 0,026 (0,019-10,383) | 0,008 (0,000-0,141) | 0,001 (0,000-0,006) | 0,013 (0,001-0,328) |
| CD3E | 0,019 (0,000-0,152) | 0,010 (0,001-0,021) | 0,039 (0,000-0,552) | 0,002 (0,000-0,159) | 0,001 (0,000-0,127) | 0,018 (0,005-0,035) | 0,123 (0,001-0,881) | 0,157 (0,000-0,298) | 0,288 (0,002-0,823) |
| CD4 | 0,004 (0,001-0,153) | 0,011 (0,003-0,016) | 0,011 (0,000-0,059) | 0,009 (0,001-0,594) | 0,002 (0,000-0,582) | 0,009 (0,007-0,020) | 0,057 (0,002-0,517) | 0,123 (0,046-4,309) | 0,304 (0,017-0,628) |
| CD8B | 0,322 (0,012-0,896) | 0,206 (0,011-0,788) | 0,447 (0,206-4,884) | 0,200 (0,042-4,269) | 0,086 (0,067-0,661) | 0,354 (0,015-2,127) | 0,105 (0,004-0,562) | 0,096 (0,046-1,711) | 0,212 (0,004-4,221) |
| CD38 | 0,006 (0,000-0,231) | 0,005 (0,003-0,070) | 0,006 (0,003-0,010) | 0,032 (0,003-0,419) | 0,048 (0,002-0,098) | 0,053 (0,003-0,064) | 0,017 (0,000-0,084) | 0,011 (0,004-0,068) | 0,009 (0,003-0,013) |
| CD44 | 1,068 (0,000-7,275) | 0,945 (0,027-1,453) | 1,951 (0,788-19,320) | 0,352 (0,101-1,330) | 0,691 (0,000-2,818) | 0,688 (0,138-18,317) | 0,931 (0,030-32,189) | 1,009 (0,513-16,656) | 1,840 (0,304-23,714) |
| CKIT | 0,003 (0,000-0,024) | 0,007 (0,001-0,199) | 0,007 (0,000-0,022) | 0,017 (0,001-1,063) | 0,003 (0,000-0,124) | 0,028 (0,008-0,080) | 0,000 (0,000-0,014) | 0,000 (0,000-0,001) | 0,000 (0,000-0,003) |
| CMYB | 0,034 (0,000-0,317) | 0,027 (0,000-0,726) | 0,082 (0,000-0,731) | 0,253 (0,064-5,990) | 0,162 (0,000-0,432) | 1,374 (0,000-5,169) | 0,005 (0,000-0,408) | 0,007 (0,000-0,083) | 0,011 (0,001-0,029) |
| CXCR4 | 0,270 (0,025-2,743) | 2,753 (0,751-5,295) | 2,049 (0,443-5,417) | 0,168 (0,012-2,786) | 0,230 (0,019-0,365) | 0,610 (0,127-8,833) | 2,022 (0,008-27,627) | 2,595 (1,340-6,639) | 4,121 (1,830-40,796) |
| EBF1 | 0,003 (0,000-6,303) | 0,000 (0,000-0,001) | 0,012 (0,000-0,135) | 0,000 (0,000-0,006) | 0,000 (0,000-0,000) | 0,000 (0,000-0,053) | 0,001 (0,000-0,148) | 0,001 (0,000-0,028) | 0,006 (0,000-0,019) |
| GATA1 | 0,000 (0,000-0,044) | 0,001 (0,000-0,101) | 0,001 (0,000-0,104) | 0,037 (0,000-0,335) | 0,008 (0,000-0,075) | 0,205 (0,000-0,827) | 0,006 (0,000-0,114) | 0,013 (0,002-0,201) | 0,002 (0,000-0,007) |
| GATA3 | 0,000 (0,000-0,005) | 0,001 (0,001-0,007) | 0,017 (0,013-0,403) | 0,003 (0,000-0,029) | 0,005 (0,002-0,019) | 0,039 (0,004-0,054) | 0,010 (0,000-0,122) | 0,023 (0,003-0,079) | 0,121 (0,047-0,217) |
| HES1 | 0,002 (0,000-0,030) | 0,002 (0,000-0,014) | 0,006 (0,000-0,049) | 0,001 (0,000-0,005) | 0,002 (0,000-0,030) | 0,003 (0,000-0,015) | 0,005 (0,001-0,046) | 0,005 (0,000-0,021) | 0,010 (0,000-0,086) |
| IKAROS | 0,033 (0,000-0,259) | 0,019 (0,001-0,707) | 0,054 (0,008-0,266) | 0,028 (0,000-0,168) | 0,024 (0,000-0,363) | 0,050 (0,016-0,291) | 0,022 (0,000-0,909) | 0,066 (0,008-0,197) | 0,040 (0,000-0,130) |
| IL7Rα | 0,005 (0,000-0,038) | 0,002 (0,000-0,004) | 0,038 (0,003-0,342) | 0,001 (0,000-0,017) | 0,002 (0,001-0,024) | 0,009 (0,001-0,718) | 0,020 (0,000-0,378) | 0,130 (0,030-0,254) | 0,432 (0,049-0,784) |
| ITGα4 | 0,039 (0,000-0,317) | 0,102 (0,000-0,199) | 0,074 (0,000-0,494) | 0,041 (0,005-0,104) | 0,025 (0,000-0,224) | 0,031 (0,000-1,488) | 0,051 (0,002-0,199) | 0,014 (0,005-0,955) | 0,092 (0,000-0,694) |
| LMO2 | 0,051 (0,007-1,046) | 0,051 (0,000-0,166) | 0,163 (0,042-6,141) | 0,061 (0,009-0,354) | 0,130 (0,000-0,218) | 0,483 (0,141-3,715) | 0,020 (0,000-0,930) | 0,140 (0,000-1,097) | 0,0470 (0,017-0,165) |
| NOTCH1 | 0,101 (0,007-1,929) | 0,035 (0,000-0,654) | 0,579 (0,018-2,414) | 0,037 (0,004-0,937) | 0,044 (0,000-0,177) | 0,130 (0,015-0,879) | 0,029 (0,000-0,385) | 0,272 (0,009-1,890) | 0,125 (0,003-0,201) |
| PSGL1 | 0,018 (0,000-0,216) | 0,017 (0,002-0,068) | 0,029 (0,000-0,121) | 0,038 (0,002-0,088) | 0,018 (0,000-0,038) | 0,043 (0,009-0,111) | 0,149 (0,001-0,858) | 0,073 (0,036-0,655) | 0,316 (0,033-0,918) |
| RAG1 | 0,324 (0,002-6,099) | 0,063 (0,024-1,321) | 0,313 (0,112-6,338) | 0,111 (0,034-1,075) | 0,127 (0,011-3,688) | 0,107 (0,000-6,130) | 0,026 (0,001-1,888) | 0,032 (0,006-0,108) | 0,084 (0,022-1,903) |
| RORC | 0,001 (0,000-0,037) | 0,000 (0,000-0,000) | 0,007 (0,000-0,110) | 0,000 (0,000-0,010) | 0,000 (0,000-0,001) | 0,000 (0,000-0,003) | 0,000 (0,000-0,006) | 0,000 (0,000-0,010) | 0,002 (0,000-0,004) |

Result of qRT-PCR on 11 healthy donors (HD), 15 patients without aGVHD or Grade I aGVHD (Gr 0-I) and 7 patients with severe aGVHD (Gr II-IV). Median (range) of relative quantity to RPL27 are indicated.
