# Supplementary material for: CXCR4-Related Increase of Circulating Human Lymphoid Progenitors after Allogeneic Hematopoietic Stem Cell Transplantation
Source: PLoS One. 2014 Mar 12;9(3):e91492. doi: 10.1371/journal.pone.0091492 (PMC3951398; doi:10.1371/journal.pone.0091492)
Supplement: Figure S2 — Absolute numbers of lymphoid progenitors correlate with aGVHD severity. (PDF) [file pone.0091492.s002.pdf]

Figure S2: Absolute numbers of lymphoid progenitors correlate with aGVHD severity

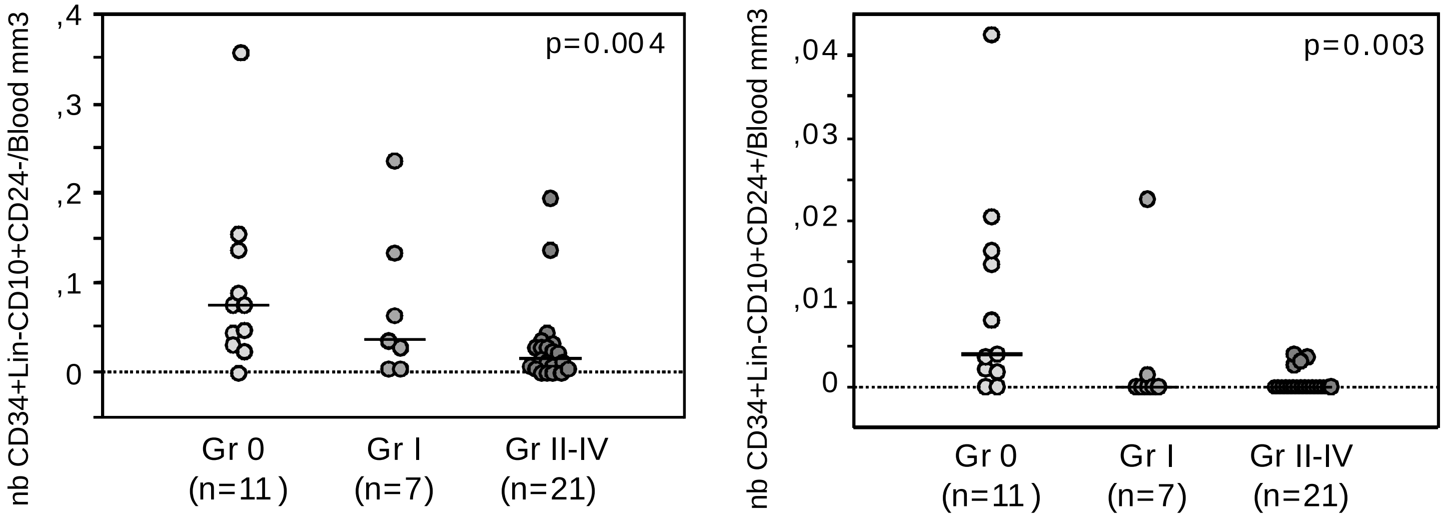

Mononuclear cells from peripheral blood from patients, 3 months after allo-HSCT, without (Gr 0) or with grade I (Gr I) or with grade II to IV (Gr II-IV) aGVHD were stained with a combination of lineage (Lin) markers and anti-CD34, anti-CD10 and anti-CD24 antibodies. There is a significant correlation between the severity of aGVHD and the number of T-committed progenitors (left) and B-committed progenitors (right) (*Jonckheere-Terpstra Test)*.
